# Variance in Brain Volume with Advancing Age: Implications for Defining the Limits of Normality

**DOI:** 10.1371/journal.pone.0084093

**Published:** 2013-12-19

**Authors:** David Alexander Dickie, Dominic E. Job, David Rodriguez Gonzalez, Susan D. Shenkin, Trevor S. Ahearn, Alison D. Murray, Joanna M. Wardlaw

**Affiliations:** 1 Brain Research Imaging Centre (BRIC), The University of Edinburgh, Neuroimaging Sciences, Western General Hospital, Edinburgh, United Kingdom; 2 Geriatric Medicine Unit, The University of Edinburgh, Royal Infirmary of Edinburgh, Edinburgh, United Kingdom; 3 Aberdeen Biomedical Imaging Centre, University of Aberdeen, Foresterhill, Aberdeen, United Kingdom; 4 Scottish Imaging Network, A Platform for Scientific Excellence (SINAPSE) collaboration, Edinburgh, United Kingdom; University of Maryland, College Park, United States of America

## Abstract

**Background:**

Statistical models of normal ageing brain tissue volumes may support earlier diagnosis of increasingly common, yet still fatal, neurodegenerative diseases. For example, the statistically defined distribution of normal ageing brain tissue volumes may be used as a reference to assess patient volumes. To date, such models were often derived from mean values which were assumed to represent the distributions and boundaries, i.e. percentile ranks, of brain tissue volume. Since it was previously unknown, the objective of the present study was to determine if this assumption was robust, i.e. whether regression models derived from mean values accurately represented the distributions and boundaries of brain tissue volume at older ages.

**Materials and Methods:**

We acquired T1-w magnetic resonance (MR) brain images of 227 normal and 219 Alzheimer’s disease (AD) subjects (aged 55-89 years) from publicly available databanks. Using nonlinear regression within both samples, we compared mean and percentile rank estimates of whole brain tissue volume by age.

**Results:**

In both the normal and AD sample, mean regression estimates of brain tissue volume often did not accurately represent percentile rank estimates (errors=-74% to 75%). In the normal sample, mean estimates generally underestimated differences in brain volume at percentile ranks *below* the mean. Conversely, in the AD sample, mean estimates generally underestimated differences in brain volume at percentile ranks *above* the mean. Differences between ages at the 5^th^ percentile rank of normal subjects were ~39% greater than mean differences in the AD subjects.

**Conclusions:**

While more data are required to make true population inferences, our results indicate that mean regression estimates may not accurately represent the distributions of ageing brain tissue volumes. This suggests that percentile rank estimates will be required to robustly define the limits of brain tissue volume in normal ageing and neurodegenerative disease.

## Introduction

Methods to assist diagnosis and prediction of common, yet still fatal, neurodegenerative diseases, such as Alzheimer’s (AD), are urgently required as the number of these cases becomes critical [1,2]. Statistical models of magnetic resonance (MR) brain imaging data may provide such a method. Specifically, models of normal brain volumes across age may highlight incipient brain tissue loss due to AD and other age-related neurodegenerative diseases [3,4]. These models are often based on central tendency, e.g. mean or median, statistical analyses. A semi-formal review (part of a wider systematic review) found that, for over 20 years, mean-based regression models have been used extensively to define normal brain volumes across age (table 1) [5].

Mean estimates may be extrapolated to define clinical distributions and boundaries if the variances in brain volume are equally Gaussian between ages and disease states. If variances are not equally Gaussian, the distributions and boundaries of ageing brain volumes may not be well represented by models based on mean estimates [6,7,8]. That is, the true range of normality and boundaries with pathology may be obscured if extrapolated from these models.

**Table 1:** Methods to Define Normal Brain Volume across Age.

**Study**	**No. of Subjects**	**Age range** ^a^	**Mean ±SD Age** ^a^	**Statistical Method** ^b^
Allen et al., 2005 [26]	87	22.0–88.0	49.4 ± 20.8	Multiple regression^M^
Courchesne et al., 2000 [27]	116	1.6–80.0	21.4 ±20.0	Regression analyses^M^
DeCarli et al., 2005 [28]	2081	34.0–96.0	62.4 ±10.4	Linear regression^M^
Fotenos et al., 2005 [18]	94	65.0–95.0	78.0 ±8.0	Hierarchical polynomial regression^M^
Ge et al., 2002 [9]	54	20.0–86.0	46.8 ±19.3	Least-squares regression^M^
Giorgio et al., 2010 [29]	66	23.0–81.6	36.7^c^	Regression analysis^M^
Good et al., 2001 [30]	465	17.0–79.0	29.5^d^	General Linear Model^M^
Gur et al., 1991 [31]	69	18.0–80.0	41.4 ±20.2	Multivariate analysis of variance^M^
Jernigan et al., 2001 [32]	78	30.0–99.0	64.0 ±17.4	Nonparametric monotone regression
Kruggel, 2006 [12]	502	16.0–70.0	30.0 ±9.6	Linear, quadratic regression^M^
Raz et al., 2005 [33]	72	20.0–77.0	52.6 ±14.1	Latent difference model^M^
Sowell et al., 2003 [34]	176	7.0–87.0	32.4 ±21.8	Quadratic multiple regression^M^
Walhovd et al., 2005 [35]	25	67.0–88.0	74.3 ±4.8	Regression analyses^M^
Ziegler et al., 2011 [36]	547	19.0–86.0	48.1 ±16.6	General linear model^M^

Note: ^M^=mean-based method; ^a^In years; ^b^This is the statistical method used to provide the majority of results, recorded as stated in the corresponding manuscript; ^c^Median; ^d^Estimated median; SD=standard deviation.

Several independent studies have shown that variance in brain tissue volume is unequal between ages [3,9,10,11,12], i.e. the range of volumes generally increases with age. This inequality of variance was removed in previous ageing brain image studies by performing data transformations, e.g. Box-Cox [12,13]. While this approach is useful in research to identify general patterns of brain ageing, it may obscure the true limits of normality and the subtle early signs of disease. We found no other previous study that attempted to define the distributions and boundaries of brain volume between ages and disease states. The true distributions of ageing brain tissue volumes and the limits of normality are therefore largely unknown. 

Specifically, it is not known whether mean (parametric) estimates of ageing brain volumes approximate percentile rank estimates. In parametric regression models it is implicitly assumed that mean estimates approximate percentile rank estimates [6]. Percentile ranks are levels that represent percentages of subjects within a distribution, e.g. the bottom 5% of subjects in a distribution have a value equal to or less than the 5^th^ percentile rank value [6]. To define these levels with mean-based regression one must assume that differences in brain tissue volume between ages are the same across all percentile ranks. For example, it must be assumed that the differences in brain tissue volume between 60 and 70 year olds at lower ranks, e.g. the 5^th^ percentile, are the same as the differences between 60 and 70 year olds at higher ranks, e.g. the 95^th^ percentile. It is unknown whether or not this is true. If it is not true then potentially important biological information may be lost by extrapolation from mean-based regression. In particular, the limits of normality and boundaries with pathology may be misrepresented [6,8]. 

In this work, using publicly available brain images, we attempted to determine whether mean regression estimates of brain volumes across age approximated percentile rank regression estimates. Our results show that, in presently available data, mean estimates generally do not approximate percentile rank estimates. We therefore suggest percentile rank models will be required to robustly define the distributions and limits of brain tissue volume in normal ageing and neurodegenerative disease.

## Materials and Methods

### Subjects

This research used publicly available imaging data not obtained at the authors’ institutions. All subjects in the Open Access Series of Imaging Studies (OASIS; http://www.oasis-brains.org/) participated in accordance with guidelines of the Washington University Human Studies Committee. All subjects in the Alzheimer's Disease Neuroimaging Initiative (ADNI; http://adni.loni.usc.edu/) provided written informed consent and were recruited with respective institutional approval.

The ADNI, through collaboration among government, private, and non-profit organizations (listed in the Financial Disclosure), recruited subjects from over 50 sites across the United States and Canada to test whether imaging and other biological markers and clinical and neuropsychological assessment can be combined to measure the progression and better treat mild cognitive impairment (MCI) and early AD. For up-to-date information, see www.adni-info.org.

MR brain images from 137 “normal” subjects (n=60, 44% female), mean age ~76 (70-89) years, were acquired from the ADNI databank (table 2). These subjects did not have dementia, but potentially had non-debilitating conditions common in ageing, e.g. hypertension. A further 90 subjects (n=65, 72% female) with similar clinical characteristics and a mean age of ~75 (60-89) years, were acquired from the OASIS databank [14] (table 2). According to a recent systematic review, ADNI and OASIS were the only public sources of structural MR brain images that had medical and cognitive metadata representative of the characteristics of normal older people (≥60 years) [5]. These subjects were reported to be representative of normal older people through cognitive and physical tests but actual measures of potentially confounding variables, e.g. blood pressure, were not available to us for all subjects [14]. We combined the ADNI and OASIS samples to create a single normal subject sample of n=227 subjects.

**Table 2:** Demographics of the ADNI and OASIS subjects.

**Sample**	**Age in years**	**No. of M:F (total)**
ADNI		
Normal	<70	0:0 (0)
	70–74	35:28 (63)
	75–79	25:22 (47)
	80–84	9:8 (17)
	85–89	8:2 (10)
	Overall	77:61 (137)
AD	<70	16:12 (28)
	70–74	14:17 (31)
	75–79	12:14 (26)
	80–84	16:8 (24)
	85–89	8:7 (15)
	Overall	66:58 (124)
OASIS		
Normal	<70	7:18 (25)
	70–74	7:19 (26)
	75–79	3:6 (9)
	80–84	4:13 (17)
	85–89	4:9 (13)
	Overall	26:72 (90)
AD	<70	6:9 (15)
	70–74	10:15 (25)
	75–79	10:13 (23)
	80–84	10:15 (25)
	85–89	3:4 (7)
	Overall	39:56 (95)

Note: No.=number; M=males; F=females; ADNI=Alzheimer’s Disease Neuroimaging Initiative; OASIS=Open Access Series of Imaging Studies.

A sample of MR brain images from 124 subjects diagnosed with AD (n=58, 47% female) and a mean age of ~75 (55-89) years was also acquired from ADNI (table 2). A further 95 AD subjects with similar demographics were acquired from OASIS (table 2) and combined with the ADNI subjects to create a single AD sample of n=219 subjects.

### MR brain image acquisition and processing

In both ADNI and OASIS, 1.5 tesla (T) magnetization prepared rapid gradient-echo (MP-RAGE) T1-weighted MR brain images were acquired in the sagittal plane at approximately 1x1x1mm resolution. The full acquisition parameters are described elsewhere [14,15].

Non-brain structure was first removed from the images, by the following steps

1. The Montreal Neurological Institute (MNI) 152 template brain mask (http://www.bic.mni.mcgill.ca/ServicesAtlases/HomePage) was fitted to each subject in two sub-steps
  - First, using Functional MRI of the Brain’s (FMRIB’s) Linear Registration Tool (FLIRT) [16], each subject was orientated to the position and angle of the template
  - Second, using Advanced Normalisation Tools (ANTS) [17], the template was diffeomorphically warped to approximate each subject’s anatomy.
2. The resulting brain mask of each subject was applied to their FLIRT registered image to remove non-brain structure.
3. The results of steps 1 and 2 were manually inspected by slice and errors, e.g. remaining skull, corrected using the Multi-image Analysis GUI (http://ric.uthscsa.edu/mango/download.html).

To be consistent with previous studies [14,18], a bias field correction was performed and grey matter (GM), white matter (WM), and cerebrospinal spinal fluid (CSF) volumes were calculated using FMRIB’s Automated Segmentation Tool (FAST) [19]. The advanced age of these subjects meant that many of them had what appeared to be WM lesions (hypointense WM regions on T1-wieghted images that are hyperintense on T2-weighted images). FAST sometimes incorrectly classified these regions as GM. The MRI sequences, e.g. fluid attenuated inversion recovery (FLAIR), required to accurately address these errors were not available for all subjects [14]. Although incorrectly classified as GM, these regions were still correctly classified as tissue, i.e. not CSF. We therefore added the GM and WM volumes within each subject to calculate overall brain tissue volume in voxels. Brain tissue volumes were then normalised (divided) by total intracranial volume (TIV=tissue+CSF). We do not report normalised CSF regression models because they were just the exact inverse of normalised whole brain tissue volume models.

#### Calculating mean and percentile rank regression models of brain volume by age

When expressed in one year intervals, there were very few subjects at some ages. This meant that it was not possible to calculate values of percentile ranks at these ages. We therefore expressed age in the following intervals: <70, 70-74, 75-79, 80-84, and 85-89 years.

Mean differences in brain tissue volume between age groups were calculated in each sample with “PROC REG” in the Statistical Analysis System (SAS) v9.3 (http://support.sas.com/documentation/cdl/en/statug/63033/HTML/default/viewer.htm#reg_toc.htm). PROC REG produces a regression equation (*y*=*βx*_*1*_ *… βx*_*n*_+*c*) to describe mean differences in a dependent variable (*y*), e.g. brain tissue volume, between values of independent variables (*x*_*1*_ *… x*_*n*_), e.g. age group. The beta (*β*) coefficients of this equation define the size of mean differences between groups.

The 5^th^, 25^th^, 50^th^, 75^th^, and 95^th^ percentile ranks of brain tissue volume were directly calculated for each age group by equation 1,

np=j+g (1.1)

$$y=1/2(x_{j}+x_{j+1})\;\;\;\;\text{if}\;g=0$$ (1.2)

$$y=x_{j+1}\;\;\;\;\;\;\;\;\;\;\;\;\;\;\;\;\;\;\;\;\text{if}\;g>0$$

where *n* is the number of subjects, for the *t*th percentile *p*=*t*/100, *j* is the integer part of *np*, *g* is the fractional part of np, *y* is the *t*th percentile, and *x*_1_, *x*_*2*_, ... , *x*_*n*_ are the ordered values of brain tissue volume. Differences in volume between ages (regression equations) at these percentile ranks (rather than the mean) were then calculated with PROC QUANTREG (http://support.sas.com/documentation/cdl/en/statug/63033/HTML/default/viewer.htm#statug_qreg_sect001.htm) in SAS. 

For both mean and percentile rank estimates, we initially performed linear regressions. These were used to produce residual plots (actual minus regression predicted volumes by age group). The linearity assumption is in question if these plots show a systematic pattern, e.g. particular age groups with skewed positive or negative residuals [6]. When the linearity assumption was in question, we performed nonlinear (cubic) regression to define differences between ages [18].

The representativeness of mean estimates was illustrated by relative percent error between the expected (mean) and observed (percentile rank) regression predictions, calculated by equation 2,

$$\frac{{\overset{⌢}{y}}_{\mu p,i}-{\overset{⌢}{y}}_{dp,i}}{\Delta_{\mu }}\times 100$$ (2)

where ${\overset{⌢}{y}}_{\mu p,i}$ is the mean-based prediction of percentile rank *p* for age group *i*, ${\overset{⌢}{y}}_{dp,i}$is the directly calculated percentile rank prediction, and Δ_*μ*_ is the overall mean change in brain tissue volume (mean regression predicted volume at <70 years minus mean regression predicted volume at 85-89 years). Due to the potential for subtle differences between ages and disease [3,18], results are reported with four significant figures.

## Results

### Mean and variance of brain volume within the normal and AD samples

**Figure 1:**
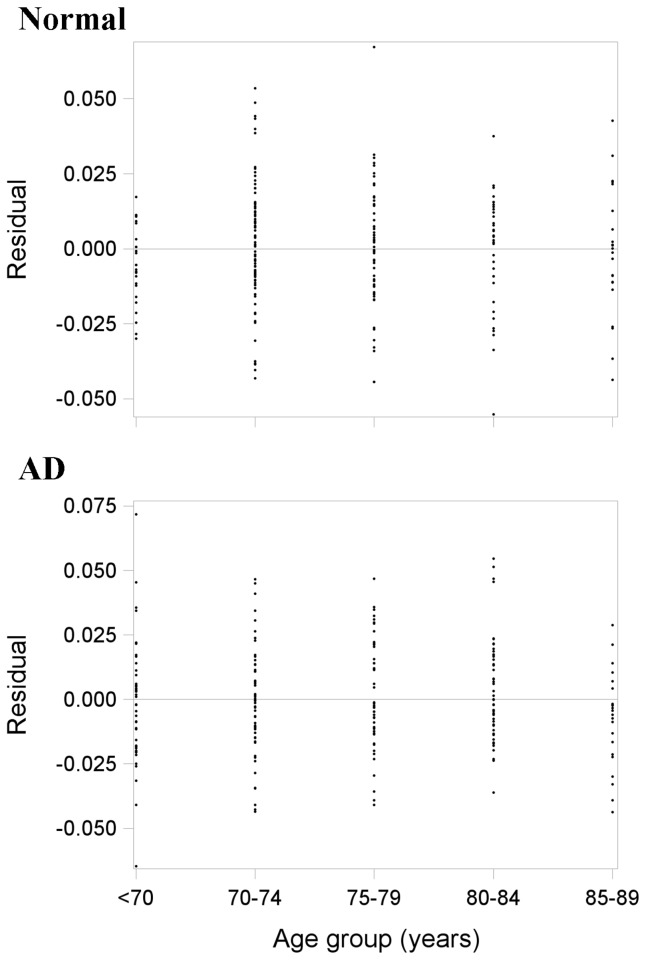
Residual plots (actual minus mean linear regression predicted brain volumes by age) from the normal (top panel; n=227) and AD (bottom panel; n=219) samples. There are skewed residuals at 70 years in the normal sample (top) and a similar pattern at 85-89 years in the AD sample (bottom). This means that the linearity assumption was in question.

The mean and standard deviation (SD) of normalised brain volume in each age group in the normal and AD samples are shown in table 3. Variance in brain tissue volume generally increased with age in the normal sample but decreased with age in the AD sample. Residual plots (actual minus regression predicted volumes by age group) from the mean linear regressions are shown in Figure 1. The different spread of points at each age group further illustrates unequal variance between ages. Moreover, skewed residuals at 70 years in the normal sample (few positive compared to negative residuals) and a similar pattern at 85-89 years in the AD sample suggested that the linearity assumption was in question. We therefore performed nonlinear regression of brain tissue volume across age in each sample.

**Table 3:** The mean and standard deviation of normalised brain volume in each age group in the normal and AD samples.

**Sample**	**Age**	**n**	**Mean**	**SD**
Normal	<70	25	0.7555	0.0130
	70-74	89	0.7569	0.0196
	75-79	56	0.749	0.0204
	80-84	34	0.7418	0.0193
	85-89	23	0.73592	0.0210
AD	<70	43	0.7399	0.0236
	70-74	56	0.7350	0.0208
	75-79	49	0.7330	0.0209
	80-84	49	0.7292	0.0199
	85-89	22	0.71285	0.0187

Note: SD=standard deviation.

### Mean and percentile rank regression estimates of brain volume across age within the normal sample

**Figure 2:**
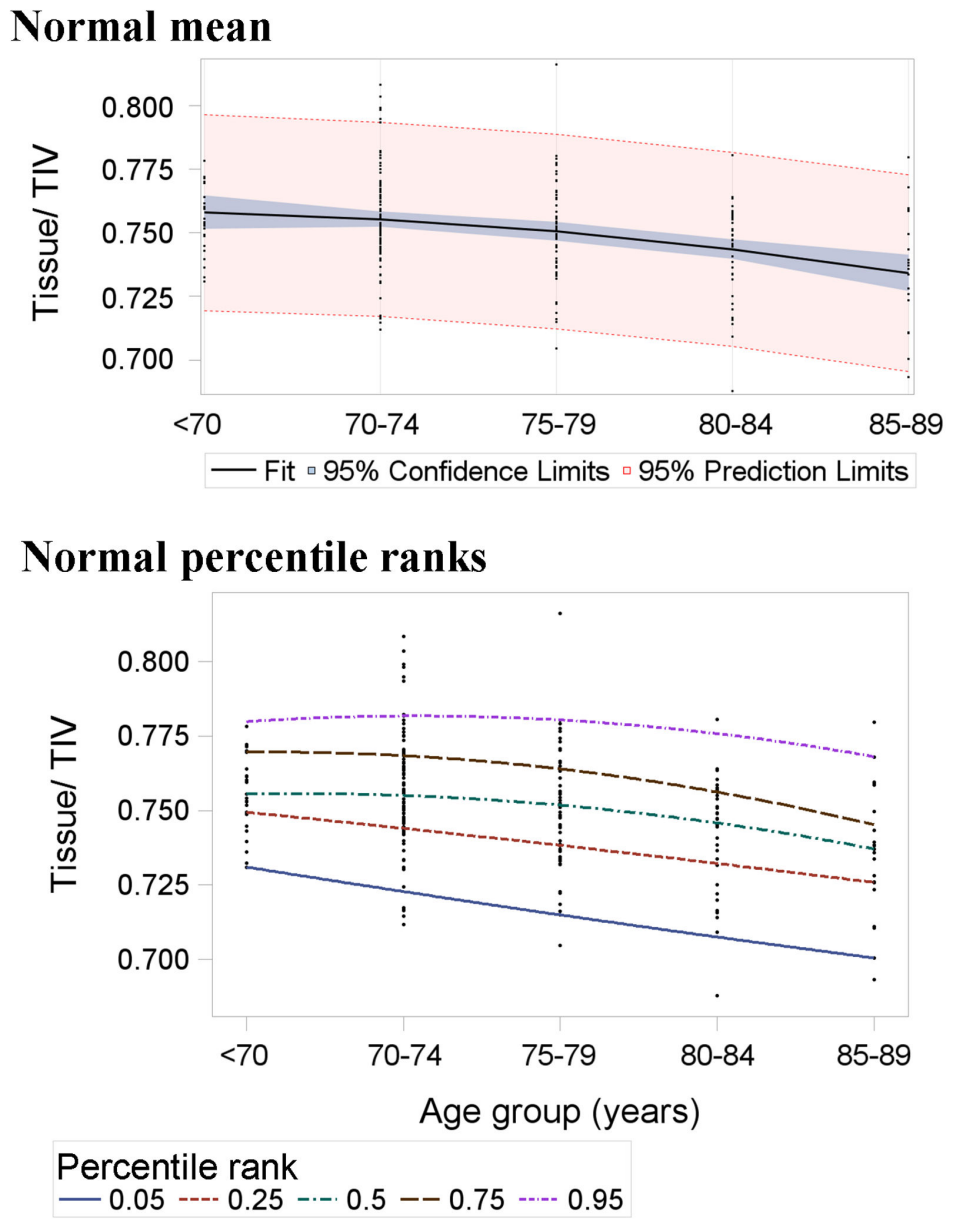
Mean (top panel) and percentile rank (bottom panel) regression estimates of brain tissue volume across age in the normal sample (n=227). The slopes of these lines represent the beta coefficients in table 4. The mean-based model expects all percentile ranks to change at the same rate, i.e. be parallel. The diverging percentile ranks show that this is not the case and that variance in brain volume generally increased with age in the normal subjects. Although some may appear linear, each line is the result of nonlinear regression (table 4).

**Figure 3:**
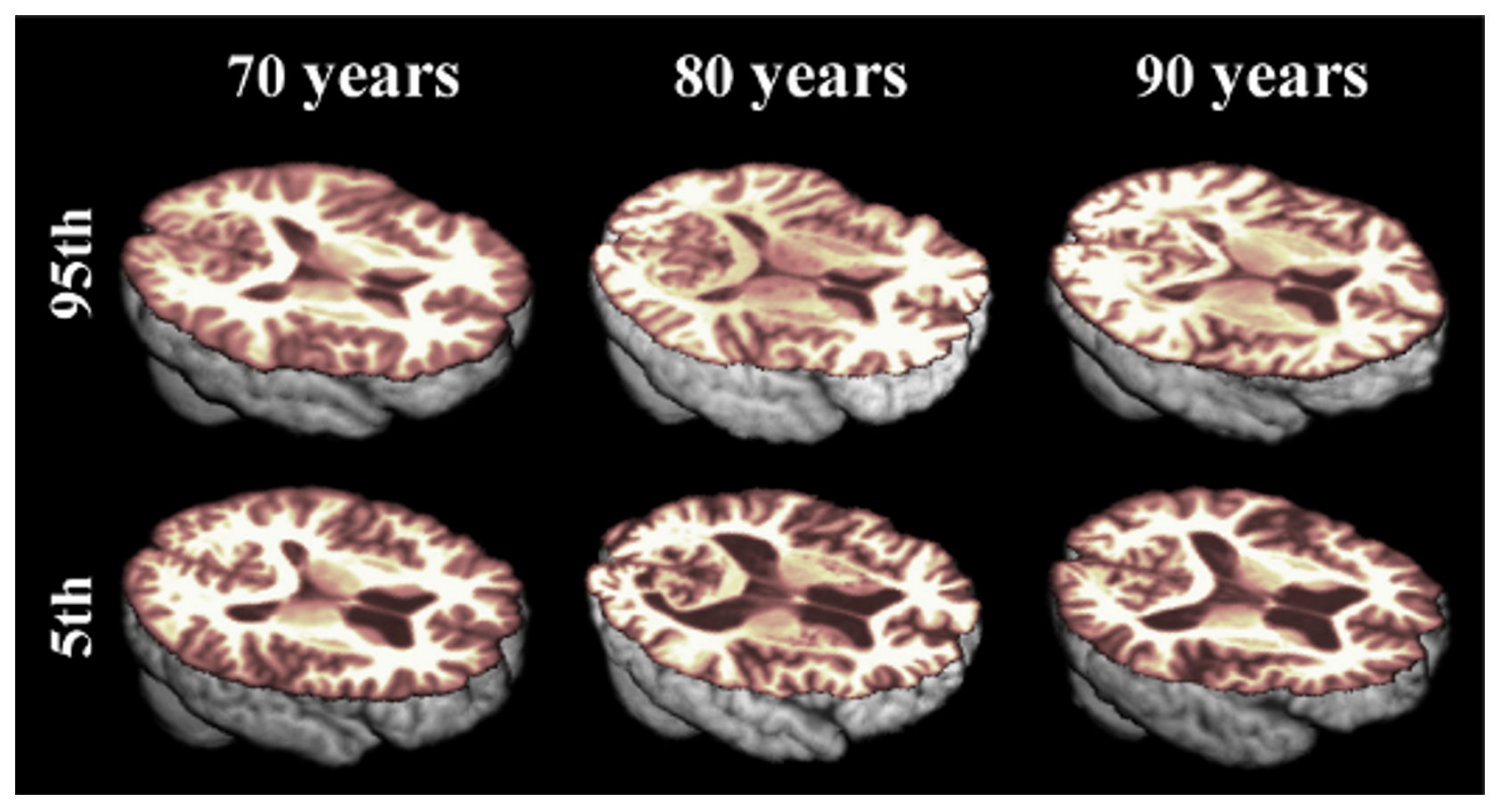
Illustration of the varying differences in normal ageing brain tissue volume, according to percentile rank. There were much greater differences between ages at the 5th percentile of brain tissue volume (bottom panel) than between ages at the 95th percentile (top panel) of normal subjects.

Mean and percentile rank regression estimates of brain volume across age in the normal sample are listed in table 4 and illustrated in figures 2 and 3. As the dashed (percentile rank) lines within each graph are generally not parallel (Figure 2), the distribution of differences in brain tissue volume between ages was not well represented by mean estimates. The diverging percentile rank lines further illustrate that variance in brain tissue volume increased with age in normal subjects. 

**Table 4:** Mean and percentile rank regression estimates of normalised brain tissue volume by age in the normal sample.

**Rank**	**Age (*x*)**	***c***	**Beta_*x***	**Beta_*x*^*2*^**	***p* prediction**	**μ prediction** (Δ_*μ*_)	**% error**
MEAN		0.7584	0.0007	-0.0011			
5^th^		0.7397	-0.0089	0.0002			
	<70 (1)				0.7310	0.7393	35
	70-74 (2)				0.7227	0.7367	59
	75-79 (3)				0.7148	0.7319	72
	80-84 (4)				0.7073	0.7249	75
	85-89 (5)				0.7002	0.7157 (0.0236)	66
25^th^		0.7545	-0.0049	-0.0002			
	<70 (1)				0.7494	0.7541	20
	70-74 (2)				0.7439	0.7515	32
	75-79 (3)				0.7380	0.7467	37
	80-84 (4)				0.7317	0.7397	34
	85-89 (5)				0.7250	0.7305 (0.0236)	23
50^th^		0.7533	0.0036	-0.0014			
	<70 (1)				0.7555	0.7529	-11
	70-74 (2)				0.7549	0.7503	-19
	75-79 (3)				0.7515	0.7455	-25
	80-84 (4)				0.7453	0.7385	-29
	85-89 (5)				0.7363	0.7293 (0.0236)	-30
75^th^		0.7674	0.0038	-0.0016			
	<70 (1)				0.7696	0.7670	-11
	70-74 (2)				0.7686	0.7644	-18
	75-79 (3)				0.7644	0.7596	-20
	80-84 (4)				0.7570	0.7526	-19
	85-89 (5)				0.7464	0.7434 (0.0236)	-13
95^th^		0.7747	0.0067	-0.0016			
	<70 (1)				0.7798	0.7743	-23
	70-74 (2)				0.7817	0.7717	-42
	75-79 (3)				0.7804	0.7669	-57
	80-84 (4)				0.7759	0.7599	-68
	85-89 (5)				0.7682	0.7507 (0.0236)	-74

Note: This table shows the regression equations for the mean and each percentile rank. Percent errors between predictions are calculated for each age relative to the overall mean regression change (Δ_*μ*_=mean regression predicted volume at <70 years minus mean regression predicted volume at 85-89 years). Positive percent errors indicate that the mean regression underestimated differences between ages, i.e. overestimated brain tissue volume at advanced ages. Negative percent errors indicate that the mean regression overestimated differences between ages, i.e. underestimated brain tissue volume at advanced ages. Age groups were coded as 1 (<70) to 5 (85-89); c=intercept; μ=mean; p=percentile rank.

 Mean estimates generally overestimated differences in brain tissue volume between ages at percentile ranks above the mean, i.e. in the upper percentile ranks, mean regression beta underestimated brain tissue volume at advanced ages. For example, the mean-based prediction of the 95^th^ percentile of brain volume at 85-89 years was short by 74% of the overall expected change between <70 and 89 years (table 4). This is in contrast to percentile ranks below the mean, where mean estimates generally underestimated differences between ages, i.e. in the lower percentile ranks, mean regression beta overestimated brain tissue volume at advanced ages. For example, the mean-based prediction of the 5^th^ percentile of brain volume at 85-89 years was inflated by 66% of the overall expected change between <70 and 89 years (table 4).

### Mean and percentile rank regression estimates of brain volume across age within the AD sample

**Figure 4:**
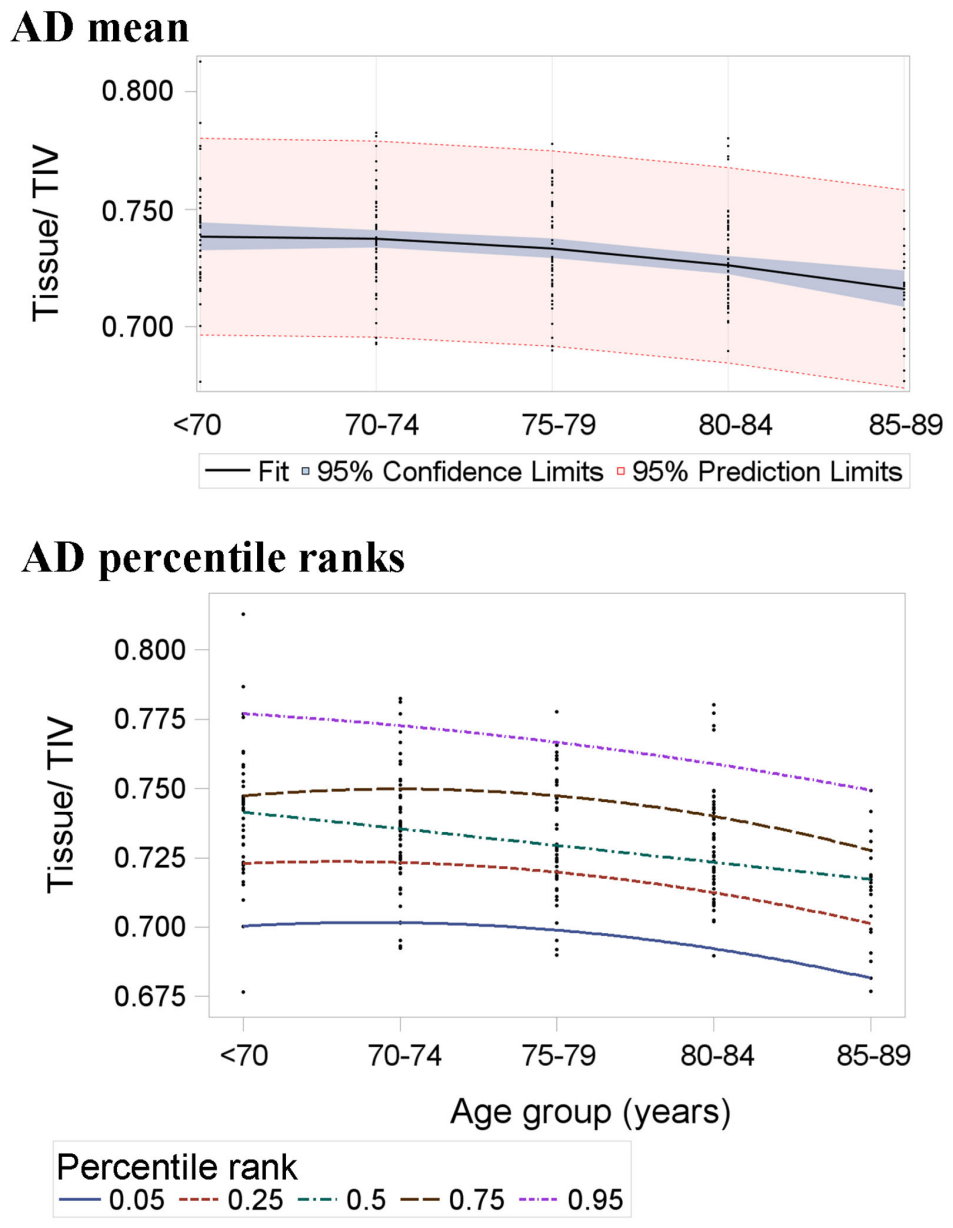
Mean (top panel) and percentile rank (bottom panel) regression estimates of brain tissue volume across age in the AD sample (n=219). The slopes of these lines represent the beta coefficients in table 5. The mean-based model expects all percentile ranks to change at the same rate, i.e. be parallel. The converging percentile ranks show that this is not the case and variance in brain volume generally decreased with age in the AD subjects. Although some may appear linear, each line is the result of nonlinear regression (table 5).

Mean and percentile rank regression estimates of brain volume across age in the AD sample are listed in table 5 and illustrated in Figure 4. As in normal subjects, the lack of parallel lines (Figure 4) shows that the distribution of differences in brain tissue volume between ages in AD was not well represented by mean estimates. The converging percentile rank lines further illustrate that variance in brain tissue volume decreased with age in AD subjects.

**Table 5:** Mean and percentile rank regression estimates of normalised brain tissue volume by age in the AD sample.

**Rank**	**Age (*x*)**	***c***	**Beta_*x***	**Beta_*x*^*2*^**	***p* prediction**	**μ prediction** (Δ_*μ*_)	**% error**
MEAN		0.73638	0.0035	-0.0015			
5^th^		0.6950	0.0072	-0.0020			
	<70 (1)				0.7002	0.6970	-14
	70-74 (2)				0.7014	0.6960	-24
	75-79 (3)				0.6986	0.6920	-30
	80-84 (4)				0.6918	0.6849	-31
	85-89 (5)				0.6810	0.6749 (0.0222)	-28
25^th^		0.7186	0.0062	-0.0019			
	<70 (1)				0.7229	0.7206	-10
	70-74 (2)				0.7234	0.7196	-17
	75-79 (3)				0.7201	0.7156	-20
	80-84 (4)				0.7130	0.7085	-20
	85-89 (5)				0.7021	0.6985 (0.0222)	-16
50^th^		0.7474	-0.0060	0.0000			
	<70 (1)				0.7414	0.7494	36
	70-74 (2)				0.7354	0.7484	59
	75-79 (3)				0.7294	0.7444	68
	80-84 (4)				0.7234	0.7373	63
	85-89 (5)				0.7174	0.7273 (0.0222)	44
75^th^		0.7399	0.0098	-0.0025			
	<70 (1)				0.7472	0.7419	-24
	70-74 (2)				0.7495	0.7409	-39
	75-79 (3)				0.7468	0.7369	-45
	80-84 (4)				0.7391	0.7298	-42
	85-89 (5)				0.7264	0.7198 (0.0222)	-30
95^th^		0.7794	-0.0016	-0.0009			
	<70 (1)				0.7769	0.7814	20
	70-74 (2)				0.7726	0.7804	35
	75-79 (3)				0.7665	0.7764	45
	80-84 (4)				0.7586	0.7693	48
	85-89 (5)				0.7489	0.7593 (0.0222)	47

Note: This table shows the regression equations for the mean and each percentile rank. Percent errors between predictions are calculated for each age relative to the overall mean regression change (Δ_*μ*_=mean regression predicted volume at <70 years minus mean regression predicted volume at 85-89 years). Positive percent errors indicate that the mean regression underestimated differences between ages, i.e. overestimated brain tissue volume at advanced ages. Negative percent errors indicate that the mean regression overestimated differences between ages, i.e. underestimated brain tissue volume at advanced ages. Age groups were coded as 1 (<70) to 5 (85-89); c=intercept; μ=mean; p=percentile rank.

Opposite to the normal sample, mean estimates generally underestimated differences in brain tissue volume between ages at percentile ranks above the mean, i.e. in the upper percentile ranks, mean regression beta overestimated brain tissue volume at advanced ages. For example, the mean-based prediction of the 95^th^ percentile of brain volume at 85-89 years was inflated by 47% of the overall expected change between <70 and 89 years (table 5). This is in contrast to percentile ranks below the mean, where mean estimates generally overestimated differences between ages, i.e. in the lower percentile ranks, mean regression beta underestimated brain tissue volume at advanced ages. For example, the mean-based prediction of the 5^th^ percentile of brain volume at 85-89 years was short by 28% of the overall expected change between <70 and 89 years (table 5).

### Comparison of normal and AD samples

There was increased overlap between normal and AD brain tissue volumes with advancing age, as illustrated by the diverging normal subject percentile ranks and converging AD subject percentile ranks (figures 2 and 4). Differences between normal ageing subjects at lower percentile ranks were similar to, or even greater than mean differences between AD subjects. For example, 5^th^ percentile rank differences in normal subjects were 38.7% greater than mean differences in AD subjects. 

## Discussion

We have shown in the publicly-available data that variances in brain tissue volume are unequal across older ages. As a result of this, the distributions and clinical limits of ageing brain tissue volumes may not be well represented by mean regression estimates. Differences in brain tissue volume between normal subjects at the lowest percentile ranks (below the mean) were considerably greater than differences at the highest percentile ranks (above the mean). For example, mean estimates inflated volumes by 35% to 75% at the 5^th^ percentile of normal subjects whereas they were short by 23% to 74% at the 95^th^ percentile of normal subjects. This normal variation needs to be adequately defined so that it is not incorrectly attributed to neurodegenerative disease. While we had insufficient data to define true population distributions, this proof of concept study suggests that percentile rank statistical models will be required to do so.

Statistical models of the normal ageing brain may be used to support earlier diagnoses of AD and related disorders [3,20,21]. Models based on mean estimates found that differences in brain tissue volume between ages were greater in AD than in normal ageing [18]. These models assume that the distributions of normal and AD brain volumes are equally Gaussian within age, and that the overlap between these distributions does not change with age [6]. We found that the distributions of brain volume were not equal within age and that the overlap between normal and AD brain volumes increased with advancing age. Further, we found that differences in brain tissue volume between normal ageing subjects at lower percentile ranks may be similar to, or even greater than differences between patients diagnosed with AD. Therefore, if a group of subjects acquired for controls in a clinical trial was unknowingly skewed to lower percentiles, true treatment effects in brain volume between normal ageing and AD may be obscured. 

A percentile rank-based reference for brain volumes may then be useful to quantitatively rank individuals or to determine if the distribution of a control group is skewed. Given the wide and irregular variance in brain volume that we have identified in a relatively small number of apparently normal subjects, this percentile rank-based reference will require much more data than are publicly available at present [5]. 

New databanks such as Minimal Interval Resonance Imaging in Alzheimer's Disease (MIRIAD), which provides longitudinal data from 23 cognitively tested normal ageing subjects [22], may help to address this shortage. The Australian Imaging Biomarkers & Lifestyle Flagship Study of Ageing (AIBL) databank is similar to ADNI and OASIS and includes 177 control subjects aged over 60 years. However, these subjects are not generally representative of the normal ageing population as they were preferentially selected as APOE ε4 allele carriers [23]. 

Other neuroimaging databank projects are ongoing or initiating that may, in due course, provide the required data. For example, we are building a brain image databank and reference atlas using existing data from >1000 cognitively tested normal subjects aged mostly between 55 and >90 years (http://www.sinapse.ac.uk/research-resources/brains-project). These data are in the process of being collated and were not available at the time of this study. In the future, by iteratively adding subjects and monitoring subsequent fluctuations in percentile rank values, we may determine the amount of data required to create robust models of brain tissue volume in normal ageing and neurodegenerative disease.

The limited number of subjects available to the present study (n=446) meant that we had to express age in five year rather than one year intervals. Differences between subjects within these intervals could not then be calculated here but could be calculated in future studies with larger samples. The age groups in this study did not have equal sample sizes and this may have contributed to unequal variance in brain volume [6]. Sample size generally decreased while variance increased with age in the normal subjects. Since a larger number of subjects generally leads to greater variance [6], inequality of variance may actually be even greater in future studies with more subjects. This further suggests that percentile rank models will be required to adequately describe the true levels and limits of ageing brain volumes. 

The inclusion of “normal” subjects with and without hypertension or other risk factors may have also contributed to the unequal variance in brain tissue volume. We could not specifically test this here as, although assessed by ADNI and OASIS, the actual measures of risk factors for all subjects were not available to us. Regardless of this, as at least 50% of subjects tend to be diagnosed with hypertension in many “normal” older cohorts [24], it could be argued that the inclusion of subjects with and without hypertension is more representative of the normal ageing population. 

The incidence of normal subjects with silent AD pathology (e.g. that might be detected with Pittsburgh Compound-B (PIB) binding) or cognitive decline that has not yet reached the point of dementia, may also partially explain the increasing variance in brain volume with age. These data were not available for all subjects and therefore a bias would have been introduced had we excluded only some subjects based on these measures. Further, it was not the aim of this study to determine the sources of normal ageing brain volume variance but to demonstrate the effect of this variance. A longitudinal study with these imaging and risk factor measures will be required to determine true population distributions and the sources of variance in normal ageing brain volumes.

Although all subjects were scanned with the same T1 MP-RAGE sequence, the different scanners used in ADNI may have also affected variance in brain volume [25]. However, all scanner protocols were standardised [15] and the magnitude of difference between brains that we detected is too large to be attributed only to differences in scanner performance. Indeed, studies using one scanner and equally sized age groups [3] have shown that irregular variance in brain tissue volume is attributable to advancing age.

Our calculation of TIV will have underestimated true TIV because the venous sinuses as well as CSF expand to occupy space vacated by the shrinking brain [24]. Further, FMRIB’s FAST may have incorrectly classified hypointense areas of WM on MP-RAGE (potentially WM lesions) as GM. The MRI sequences, e.g. FLAIR, required to correct these errors were not available for all subjects [14]. These regions were still correctly classified as tissue, i.e. not CSF, and so we combined GM and WM volumes to calculate whole brain tissue volume for each subject. Future studies and brain image databanks will need to acquire additional sequences, such as FLAIR, for more accurate calculations of brain and specific tissue, i.e. GM vs. WM, volumes.

Percentile rank brain volume models may provide at least two novel benefits. The first is that, given the general association between brain volume and cognitive function [18], percentile ranks may provide a measure to predict future brain loss and cognitive decline in individual patients. For example, normal subjects at the lowest percentile ranks may be at greater risk of developing cognitive decline and dementia. We will test this in a planned longitudinal study. The second benefit is that percentile ranks provide more detailed descriptions of the differences between normal and diseased groups. That is, they show whether general (mean) differences are due to consistent differences between subject groups or whether one group has a skewed distribution of brain structure, e.g. a proportion of subjects with extremely low values of brain volume. These benefits suggest that percentile rank models may ultimately provide deeper understanding of brain volume changes in normal ageing and, in future, assist diagnoses of neurodegenerative disease.

## References

[1] Selkoe DJ (2001) Alzheimer's disease: genes, proteins, and therapy. Physiol Rev 81: 741-766. PubMed: 11274343. 11274343 10.1152/physrev.2001.81.2.741

[2] Selkoe DJ (2013) The Therapeutics of Alzheimer’s Disease: Where We Stand and Where We are Heading Ann Neurol Accepted Article ( Accepted, unedited articles published online and citable. The final edited and typeset version of record will appear in future): page numbers not yet assigned. 10.1002/ana.24001 25813842

[3] Farrell C, Chappell F, Armitage PA, Keston P, MacLullich A et al. (2009) Development and initial testing of normal reference MR images for the brain at ages 65–70 and 75–80 years. Eur Radiol 19: 177-183. doi:10.1007/s00330-008-1119-2. PubMed: 18690455. 18690455

[4] Fox NC, Schott JM (2004) Imaging cerebral atrophy: normal ageing to Alzheimer's disease. Lancet 363: 392-394. doi:10.1016/S0140-6736(04)15441-X. 15074306

[5] Dickie DA, Job DE, Poole I, Ahearn TS, Staff RT et al. (2012) Do brain image databanks support understanding of normal ageing brain structure? A systematic review. Eur Radiol 22: 1385-1394. doi:10.1007/s00330-012-2392-7. PubMed: 22354559. 22354559

[6] Freedman D, Pisani R, Purves R (2007) Statistics. New York: WW Norton.

[7] Koenker R, Bassett Jr G (1978) Regression quantiles. Econometrica: Journal of the Econometric Society 46: 33-50. doi:10.2307/1913643.

[8] Elveback LR, Guillier CL, Keating Jr FR (1970) Health, Normality, and the Ghost of Gauss. JAMA 211: 69-75. doi:10.1001/jama.1970.03170010023004. PubMed: 5466893. 5466893

[9] Ge Y, Grossman RI, Babb JS, Rabin ML, Mannon LJ et al. (2002) Age-related total gray matter and white matter changes in normal adult brain. Part I: volumetric MR imaging analysis. AJNR Am J Neuroradiol 23: 1327-1333. PubMed: 12223373. 12223373 PMC7976241

[10] Manolio TA, Kronmal RA, Burke GL, Poirier V, O'Leary DH et al. (1994) Magnetic resonance abnormalities and cardiovascular disease in older adults. The Cardiovascular Health Study. Stroke 25: 318-327. doi:10.1161/01.STR.25.2.318. PubMed: 8303738. 8303738

[11] Resnick SM, Pham DL, Kraut MA, Zonderman AB, Davatzikos C (2003) Longitudinal magnetic resonance imaging studies of older adults: a shrinking brain. J Neurosci 23: 3295-3301. PubMed: 12716936. 12716936 10.1523/JNEUROSCI.23-08-03295.2003 PMC6742337

[12] Kruggel F (2006) MRI-based volumetry of head compartments: normative values of healthy adults. NeuroImage 30: 1-11. doi:10.1016/j.neuroimage.2005.09.063. PubMed: 16289929. 16289929

[13] Box GEP, Cox DR (1964) An Analysis of Transformations. Journal of the Royal Statistical Society Series B Statistical Methodology) 26: 211-252.

[14] Marcus DS, Wang TH, Parker J, Csernansky JG, Morris JC et al. (2007) Open Access Series of Imaging Studies (OASIS): Cross-sectional MRI Data in Young, Middle Aged, Nondemented, and Demented Older Adults. J Cogn Neurosci 19: 1498-1507. doi:10.1162/jocn.2007.19.9.1498. PubMed: 17714011. 17714011

[15] Jack Jr CR, Bernstein MA, Fox NC, Thompson P, Alexander G et al. (2008) The Alzheimer's disease neuroimaging initiative (ADNI): MRI methods. J Magn Reson Imaging 27: 685-691. doi:10.1002/jmri.21049. PubMed: 18302232. 18302232 PMC2544629

[16] Jenkinson M, Smith S (2001) A global optimisation method for robust affine registration of brain images. Med Image Anal 5: 143-156. doi:10.1016/S1361-8415(01)00036-6. PubMed: 11516708. 11516708

[17] Avants BB, Epstein CL, Grossman M, Gee JC (2008) Symmetric diffeomorphic image registration with cross-correlation: Evaluating automated labeling of elderly and neurodegenerative brain. Med Image Anal 12: 26-41. doi:10.1016/j.media.2007.06.004. PubMed: 17659998. 17659998 PMC2276735

[18] Fotenos AF, Snyder AZ, Girton LE, Morris JC, Buckner RL (2005) Normative estimates of cross-sectional and longitudinal brain volume decline in aging and AD. Neurology 64: 1032-1039. doi:10.1212/01.WNL.0000154530.72969.11. PubMed: 15781822. 15781822

[19] Zhang Y, Brady M, Smith S (2001) Segmentation of brain MR images through a hidden Markov random field model and the expectation-maximization algorithm. IEEE Trans Med Imaging 20: 45-57. doi:10.1109/42.906424. PubMed: 11293691. 11293691

[20] Fox NC, Crum WR, Scahill RI, Stevens JM, Janssen JC et al. (2001) Imaging of onset and progression of Alzheimer's disease with voxel-compression mapping of serial magnetic resonance images. Lancet 358: 201-205. doi:10.1016/S0140-6736(01)05408-3. PubMed: 11476837. 11476837

[21] McEvoy LK, Fennema-Notestine C, Roddey JC, Hagler DJ, Holland D et al. (2009) Alzheimer Disease: Quantitative Structural Neuroimaging for Detection and Prediction of Clinical and Structural Changes in Mild Cognitive Impairment. Radiology 251: 195-205. doi:10.1148/radiol.2511080924. PubMed: 19201945. 19201945 PMC2663582

[22] Malone IB, Cash D, Ridgway GR, MacManus DG, Ourselin S et al. (2013) MIRIAD—Public release of a multiple time point Alzheimer's MR imaging dataset. NeuroImage 70: 33-36. doi:10.1016/j.neuroimage.2012.12.044. PubMed: 23274184. 23274184 PMC3809512

[23] Ellis KA, Rowe CC, Villemagne VL, Martins RN, Masters CL et al. (2010) Addressing population aging and Alzheimer's disease through the Australian Imaging Biomarkers and Lifestyle study: Collaboration with the Alzheimer's Disease Neuroimaging Initiative. Alzheimers Dement 6: 291-296. doi:10.1016/j.jalz.2010.05.958. PubMed: 20451879. 20451879

[24] Aribisala BS, Valdés Hernández MC, Royle NA, Morris Z, Muñoz Maniega S, et al. (2012) Brain atrophy associations with white matter lesions in the ageing brain: the Lothian Birth Cohort 1936. Eur Radiol: doi:10.1007/s00330-00012-02677-x. 23114884

[25] Jovicich J, Czanner S, Han X, Salat D, van der Kouwe A et al. (2009) MRI-derived measurements of human subcortical, ventricular and intracranial brain volumes: Reliability effects of scan sessions, acquisition sequences, data analyses, scanner upgrade, scanner vendors and field strengths. NeuroImage 46: 177-192. doi:10.1016/j.neuroimage.2009.02.010. PubMed: 19233293. 19233293 PMC2866077

[26] Allen JS, Bruss J, Brown CK, Damasio H (2005) Normal neuroanatomical variation due to age: the major lobes and a parcellation of the temporal region. Neurobiol Aging 26: 1245-1260. doi:10.1016/j.neurobiolaging.2005.05.023. PubMed: 16046030. 16046030

[27] Courchesne E, Chisum HJ, Townsend J, Cowles A, Covington J et al. (2000) Normal Brain Development and Aging: Quantitative Analysis at in Vivo MR Imaging in Healthy Volunteers. Radiology 216: 672-682. doi:10.1148/radiology.216.3.r00au37672. PubMed: 10966694. 10966694

[28] DeCarli C, Massaro J, Harvey D, Hald J, Tullberg M et al. (2005) Measures of brain morphology and infarction in the Framingham Heart Study: establishing what is normal. Neurobiol Aging 26: 491-510. doi:10.1016/j.neurobiolaging.2004.05.004. PubMed: 15653178. 15653178

[29] Giorgio A, Santelli L, Tomassini V, Bosnell R, Smith S et al. (2010) Age-related changes in grey and white matter structure throughout adulthood. NeuroImage 51: 943-951. doi:10.1016/j.neuroimage.2010.03.004. PubMed: 20211265. 20211265 PMC2896477

[30] Good CD, Johnsrude IS, Ashburner J, Henson RNA, Friston KJ et al. (2001) A Voxel-Based Morphometric Study of Ageing in 465 Normal Adult Human Brains. NeuroImage 14: 21-36. doi:10.1006/nimg.2001.0786. PubMed: 11525331. 11525331

[31] Gur RC, Mozley PD, Resnick SM, Gottlieb GL, Kohn M et al. (1991) Gender differences in age effect on brain atrophy measured by magnetic resonance imaging. Proc Natl Acad Sci U S A 88: 2845-2849. doi:10.1073/pnas.88.7.2845. PubMed: 2011592. 2011592 PMC51336

[32] Jernigan TL, Archibald SL, Fennema-Notestine C, Gamst AC, Stout JC et al. (2001) Effects of age on tissues and regions of the cerebrum and cerebellum. Neurobiol Aging 22: 581-594. doi:10.1016/S0197-4580(01)00217-2. PubMed: 11445259. 11445259

[33] Raz N, Lindenberger U, Rodrigue KM, Kennedy KM, Head D et al. (2005) Regional Brain Changes in Aging Healthy Adults: General Trends, Individual Differences and Modifiers. Cereb Cortex 15: 1676-1689. doi:10.1093/cercor/bhi044. PubMed: 15703252. 15703252

[34] Sowell ER, Peterson BS, Thompson PM, Welcome SE, Henkenius AL et al. (2003) Mapping cortical change across the human life span. Nat Neurosci 6: 309-315. doi:10.1038/nn1008. PubMed: 12548289. 12548289

[35] Walhovd KB, Fjell AM, Reinvang I, Lundervold A, Dale AM et al. (2005) Effects of age on volumes of cortex, white matter and subcortical structures. Neurobiol Aging 26: 1261-1270. doi:10.1016/j.neurobiolaging.2005.05.020. PubMed: 16005549. 16005549

[36] Ziegler G, Dahnke R, Jäncke L, Yotter RA, May A et al. (2012) Brain structural trajectories over the adult lifespan. Hum Brain Mapp 33: 2377–2389. PubMed: 21898677. 21898677 10.1002/hbm.21374 PMC6870331

