# Correction: Variance in Brain Volume with Advancing Age: Implications for Defining the Limits of Normality

**DOI:** 10.1371/annotation/21fb1298-a831-423f-a247-205641dda40c

**Published:** 2014-01-16

**Authors:** David Alexander Dickie, Dominic E. Job, David Rodriguez Gonzalez, Susan D. Shenkin, Trevor S. Ahearn, Alison D. Murray, Joanna M. Wardlaw

In the Funding statement, multiple funding organizations and grants are incorrectly omitted.

The following paragraph should be read with the Funding statement:

"The authors would like to acknowledge and thank the following centres and funders. This work was carried out in The University of Edinburgh Brain Research Imaging Centre (BRIC; http://www.bric.ed.ac.uk/) [^] and the University of Aberdeen Biomedical Imaging Centre (http://www.abdn.ac.uk/ims/imaging/). [^] Both centres are part of the Scottish Imaging Network, A Platform for Scientific Excellence (SINAPSE)collaboration (http://www.sinapse.ac.uk/), [^] funded by the Scottish Funding Council, Scottish Executive Chief Scientist Office, and the six collaborator Universities. Professor Joanna M. Wardlaw was funded by the Scottish Funding Council and Scottish Executive Chief Scientist Office through the SINAPSE collaboration. David Alexander Dickie was funded by a SINAPSE industrial collaboration (SPIRIT) PhD scholarship with TMVSE, a Medical Research Council (MRC) scholarship, and the Tony Watson Scholarship bequest to The University of Edinburgh. Dr Dominic E. Job was funded by Wellcome Trust Grant 007393/Z/05/Z. Dr Trevor S. Ahearn was funded by SINAPSE and the University of Aberdeen. Professor Alison D. Murray was funded by NHS Grampian via the University of Aberdeen."

